# Clinical and Metabolic Associations of Metabolic Dysfunction-Associated Steatotic Liver Disease in Young Adults: A Cross-Sectional Analysis

**DOI:** 10.7759/cureus.115439

**Published:** 2026-08-30

**Authors:** Prashant Pandey, Abhijeet Rai, Ashish Jangid, Madhukar Rai, Ashutosh Shrivastava, Mohammad Shafat Siddiqui, Ayub Khan, Akash Marade, Abha Pandey, Shobhit Agarwal

**Affiliations:** 1 General Medicine, Heritage Institute of Medical Sciences (HIMS), Varanasi, IND; 2 Gastroenterology, Heritage Institute of Medical Sciences (HIMS), Varanasi, IND; 3 Community Medicine, Heritage Institute of Medical Sciences (HIMS), Varanasi, IND; 4 Life Sciences, Galgotias University, Noida, IND

**Keywords:** controlled attenuation parameter, dyslipidaemia, fibroscan, liver stiffness measurement, metabolic dysfunction-associated steatotic liver disease (masld), metabolic syndrome, obesity, transient elastography, young adults

## Abstract

Background: Metabolic dysfunction-associated steatotic liver disease (MASLD) is increasingly recognized as the hepatic manifestation of systemic metabolic dysfunction and is closely associated with obesity, dyslipidaemia, insulin resistance, and metabolic syndrome. However, evidence regarding its burden and metabolic correlates among apparently healthy young adults remains limited. This study evaluated the prevalence of MASLD and its association with clinical, anthropometric, metabolic, and hepatic parameters in a young adult population.

Methods: This hospital-based cross-sectional study included 154 apparently healthy adults aged 20-40 years. Clinical evaluation, anthropometric measurements, biochemical investigations, abdominal ultrasonography, and transient elastography (FibroScan®) were performed. Hepatic steatosis was assessed using ultrasonography and controlled attenuation parameter (CAP), while liver fibrosis was evaluated using liver stiffness measurement (LSM) and the aspartate aminotransferase (AST)-to-platelet ratio index (APRI). Participants were categorized into MASLD and non-MASLD groups, and comparisons were performed using appropriate statistical tests.

Results: Ultrasonographic evidence of MASLD was observed in 86 participants (55.8%). Compared with the non-MASLD group, participants with MASLD had significantly higher systolic and diastolic blood pressure, body weight, waist circumference, hip circumference, body mass index, waist-to-hip ratio, total cholesterol, low-density lipoprotein cholesterol, triglycerides, AST, alanine aminotransferase (ALT), APRI score, CAP, and LSM (all *p* < 0.05). Fasting plasma glucose, fasting insulin, glycated haemoglobin, homeostasis model assessment for insulin resistance (HOMA-IR), HOMA for beta cell function (HOMA-β), and high-density lipoprotein (HDL) cholesterol did not differ significantly between the groups.

Conclusion: MASLD was highly prevalent among apparently healthy young adults and was significantly associated with increased adiposity, adverse lipid profiles, elevated liver enzymes, and greater hepatic steatosis and fibrosis. These findings demonstrate significant associations between MASLD and adverse anthropometric, lipid, biochemical, and non-invasive hepatic parameters in this young adult cohort. Prospective multicentre studies are warranted to determine the temporal and clinical significance of these associations.

## Introduction

Metabolic dysfunction-associated steatotic liver disease (MASLD), formerly termed non-alcoholic fatty liver disease (NAFLD), is the most common chronic liver disease worldwide and has emerged as a major public health challenge owing to its strong association with obesity, insulin resistance, type 2 diabetes mellitus (T2DM), dyslipidaemia, and cardiovascular disease. The recent transition from NAFLD to MASLD reflects a paradigm shift toward recognising metabolic dysfunction as the principal driver of hepatic steatosis and better aligns the disease nomenclature with its underlying pathophysiology and clinical spectrum [1-4]. Although frequently asymptomatic during its early stages, MASLD contributes substantially to liver-related complications, cardiovascular morbidity, and all-cause mortality [3-6].

Recent estimates indicate that MASLD affects approximately one-third of the global adult population, with prevalence continuing to rise in parallel with increasing rates of obesity and metabolic syndrome [3,4]. In India, the burden of MASLD has increased considerably over the past decade, with community- and hospital-based studies reporting prevalence rates ranging from 25% to 38% [7,8]. The disease is increasingly recognised among individuals without obesity, particularly in Asian populations, where increased visceral adiposity and insulin resistance occur despite relatively normal body mass index (BMI) [7-11].

MASLD is now regarded as the hepatic manifestation of systemic metabolic dysfunction rather than an isolated liver disorder. Insulin resistance plays a central role in disease pathogenesis by promoting adipose tissue lipolysis, increased hepatic free fatty acid influx, enhanced de novo lipogenesis, and impaired fatty acid oxidation, resulting in hepatic lipid accumulation and progressive liver injury [12-18]. Persistent metabolic stress further induces oxidative stress, mitochondrial dysfunction, chronic low-grade inflammation, and fibrogenesis, contributing to disease progression from simple steatosis to steatohepatitis, advanced fibrosis, cirrhosis, and hepatocellular carcinoma [12-13,18].

Emerging evidence indicates that the relationship between MASLD and metabolic syndrome is bidirectional. While metabolic syndrome predisposes individuals to hepatic steatosis, longitudinal studies have demonstrated that MASLD independently predicts the subsequent development of T2DM, cardiovascular disease, and metabolic syndrome [19-21]. Consequently, MASLD is increasingly recognised as being closely associated with systemic metabolic dysfunction and cardiometabolic risk [18-21]. This concept is especially relevant in South Asian populations, who develop insulin resistance and cardiometabolic complications at lower levels of adiposity than Western populations [22-26].

Accurate non-invasive assessment of hepatic steatosis and fibrosis is essential for identifying individuals at increased risk of disease progression. Conventional ultrasonography remains the most widely used imaging modality for detecting hepatic steatosis because of its accessibility and affordability. However, transient elastography (FibroScan) enables quantitative assessment of hepatic steatosis using the controlled attenuation parameter (CAP) and simultaneous evaluation of liver fibrosis through liver stiffness measurement, providing a reliable, non-invasive alternative to liver biopsy for risk stratification [5, 27,28]. Combining these imaging modalities with anthropometric indices and metabolic biomarkers, including fasting insulin and the homeostatic model assessment of insulin resistance (HOMA-IR), may improve early identification of individuals at increased cardiometabolic risk [16,27,28].

Although numerous studies have evaluated MASLD in individuals with obesity, T2DM, or established metabolic syndrome, evidence regarding its role as an early marker of metabolic dysfunction among apparently healthy young adults remains limited, particularly in the Indian population. Furthermore, few studies have simultaneously evaluated ultrasonographic findings, transient elastography-derived steatosis and fibrosis, insulin resistance, and metabolic risk factors within the same cohort. Addressing this knowledge gap is important because early identification of metabolically at-risk individuals may facilitate timely lifestyle interventions and reduce the future burden of hepatic and cardiovascular disease.

Therefore, the present study was undertaken to evaluate the prevalence of MASLD and its association with anthropometric, clinical, metabolic, biochemical, and non-invasive hepatic parameters, including measures of hepatic steatosis and fibrosis, among apparently healthy young adults attending a tertiary care teaching hospital.

## Materials and methods

This hospital-based cross-sectional study was conducted in the Departments of General Medicine and Radiodiagnosis at Heritage Institute of Medical Sciences, a tertiary care teaching hospital in Varanasi, Uttar Pradesh, India, between September 2024 and March 2026. The study aimed to investigate the prevalence of MASLD and its association with metabolic syndrome and related clinical and metabolic risk factors among apparently healthy young adults. The study protocol was approved by the Institutional Ethics Committee of the participating institution (approval no. HIMS/IEC/206/2024), and written informed consent was obtained from all participants before enrolment. The study was conducted in accordance with the ethical principles of the Declaration of Helsinki.

Apparently healthy undergraduate and postgraduate medical students aged 20-40 years were recruited using a consecutive sampling approach. Individuals with a known history of diabetes mellitus, chronic liver disease, significant alcohol consumption was assessed using a structured questionnaire recording the type, quantity, and frequency of alcohol intake (defined as >20 g/day for men and >10 g/day for women), use of hepatotoxic medications, or previously diagnosed hypertension, dyslipidaemia, or cardiovascular disease were excluded from the study.

The sample size was calculated using the formula for comparison of two proportions, assuming a prevalence of metabolic syndrome of 57.8% among individuals with fatty liver disease and 22% among healthy controls, with 95% confidence and 90% power. After applying a design effect of two, the minimum required sample size was 68 participants per group. A total of 154 eligible participants were enrolled using consecutive sampling.

Demographic characteristics, medical history, and lifestyle-related information were obtained using a predesigned structured questionnaire. Anthropometric measurements, including height, weight, waist circumference, and hip circumference, were recorded by trained investigators following standardized protocols. Blood pressure and pulse rate were measured after an adequate period of rest using calibrated instruments. Body mass index (BMI) was calculated as weight (kg)/height (m²) and categorized according to the World Health Organization (WHO) recommendations for Asian populations, while waist-to-hip ratio (WHR) was calculated as the ratio of waist circumference to hip circumference [22].

Hepatic steatosis was evaluated by abdominal ultrasonography performed by an experienced radiologist using standardized diagnostic criteria and graded from Grade 0 to Grade III according to the severity of steatosis [5]. Ultrasonographic hepatic steatosis was graded as follows: Grade 0, normal hepatic echogenicity; Grade I, mild steatosis with a slight diffuse increase in hepatic echogenicity and preserved visualization of the portal vein walls and diaphragm; Grade II, moderate steatosis with increased hepatic echogenicity and partial obscuration of the portal vein walls and diaphragm; and Grade III, severe steatosis with marked increase in hepatic echogenicity and poor or absent visualization of the portal vein walls and diaphragm. MASLD was defined as hepatic steatosis detected by ultrasonography in the presence of at least one cardiometabolic risk factor. Cardiometabolic risk factors were defined as BMI ≥23 kg/m² for overweight, BMI ≥25 kg/m² for obesity, HOMA-IR >2.5 for insulin resistance, or fasting insulin >15 µIU/mL for elevated fasting insulin. Participants with ultrasonographic evidence of hepatic steatosis who fulfilled at least one of these criteria were classified as having MASLD. Because these cardiometabolic characteristics were incorporated into the operational definition of MASLD, subsequent analyses examining these same variables between MASLD and non-MASLD groups were considered exploratory and were interpreted as associations rather than independent predictors.

Liver steatosis and fibrosis were further assessed using transient elastography (FibroScan®, Echosens, Paris, France) after an overnight fast. The appropriate FibroScan probe (M or XL as applicable) was selected according to participant characteristics. At least 10 valid measurements were obtained for each participant. Examinations were considered reliable when at least 10 valid measurements were obtained and the interquartile range (IQR)-to-median ratio for liver stiffness measurement (LSM) was ≤30%. Hepatic steatosis was quantified using the CAP, expressed in dB/m, and categorized as S0 (<248 dB/m, no steatosis), S1 (248-267 dB/m, mild steatosis), S2 (268-279 dB/m, moderate steatosis), and S3 (≥280 dB/m, severe steatosis). Liver fibrosis was assessed using liver stiffness measurement (LSM), expressed in kPa, and staged as F0 (LSM <2.5 kPa), F1 (LSM 2.5-7kPa), F2 (LSM 7.0-9.5kPa), F3 (LSM 9.5-12.5 kPa), F4 (LSM >12.5 kPa), based on established transient elastography criteria [5,27,28]. Higher CAP values indicated greater hepatic steatosis, whereas higher LSM values indicated greater liver stiffness and a greater likelihood of hepatic fibrosis.

Venous blood samples were collected after an overnight fast of 8-12 hours for biochemical analysis. Fasting plasma glucose, fasting serum insulin, glycated haemoglobin (HbA1c), lipid profile, aspartate aminotransferase (AST), alanine aminotransferase (ALT), platelet count, and viral serological markers were measured using standard laboratory methods. Insulin resistance and pancreatic β-cell function were estimated using the Homeostasis Model Assessment indices (HOMA-IR and HOMA-β) [16]. The AST-to-platelet ratio index (APRI) was calculated using the standard formula: APRI = [(AST level/upper limit of normal AST) × 100] / platelet count (10⁹/L). Higher APRI values indicate a greater likelihood of hepatic fibrosis [27,28]. Metabolic syndrome was diagnosed according to the National Cholesterol Education Program Adult Treatment Panel III (NCEP ATP III) criteria [29].

Data were analysed using IBM SPSS Statistics version 26.0 (IBM Corp., Armonk, NY, USA). Continuous variables were expressed as mean ± standard deviation or median (interquartile range), while categorical variables were presented as frequencies and percentages. Group comparisons were performed using the independent-samples Student's t-test or Mann-Whitney U test for continuous variables and the chi-square test or Fisher's exact test for categorical variables, as appropriate. Point-biserial correlation analysis was performed to evaluate the relationship between MASLD status and continuous clinical, anthropometric, metabolic, and hepatic parameters. A two-tailed P value <0.05 was considered statistically significant.

## Results

A total of 154 apparently healthy young adults were included in the analysis. Of the 154 participants, 68 (44.2%) were female, and 86 (55.8%) were male. Among participants without MASLD, 32 (47.1%) were female, and 36 (52.9%) were male, whereas among those with MASLD, 36 (41.9%) were female and 50 (58.1%) were male. The baseline demographic, clinical, and anthropometric characteristics of participants stratified according to MASLD status are presented in Table 1. The mean age was comparable between the participants with and without MASLD (23 ± 3 years in both groups; p ≥ 0.05). Similarly, no significant differences were observed in pulse rate or height between the two groups (p ≥ 0.05).

**Table 1:** Comparison of demographic, anthropometric, and clinical characteristics between the participants with and without MASLD (N = 154). Values are expressed as mean ± standard deviation. An independent-samples Student's t-test was used to compare continuous variables between the non-metabolic dysfunction-associated steatotic liver disease (MASLD) and MASLD groups. A two-tailed P < 0.05 was considered statistically significant. Abbreviations: SBP, systolic blood pressure; DBP, diastolic blood pressure; BMI, body mass index; WHR, waist-to-hip ratio

Parameter	No MASLD (n = 68)	MASLD (n = 86)	Test statistic (t)	P-value
Age (years)	23.10 ± 3.04	23.30 ± 2.94	−0.412	0.681
SBP (mmHg)	117.65 ± 9.27	121.58 ± 9.30	−2.610	0.010
DBP (mmHg)	78.26 ± 8.92	81.77 ± 8.07	−2.553	0.012
Pulse rate (beats/min)	80.35 ± 7.31	81.22 ± 8.21	−0.684	0.495
Height (cm)	167.87 ± 11.80	169.09 ± 10.96	−0.666	0.506
Weight (kg)	64.25 ± 14.78	70.67 ± 13.15	−2.848	0.005
Waist circumference (cm)	82.65 ± 10.18	88.74 ± 9.96	−3.735	<0.001
Hip circumference (cm)	95.07 ± 8.36	98.74 ± 9.36	−2.532	0.012
BMI (kg/m²)	22.60 ± 3.39	24.63 ± 3.30	−3.748	<0.001
WHR	0.87 ± 0.08	0.90 ± 0.08	−2.342	0.020

Participants with MASLD had significantly higher systolic blood pressure (SBP) (121.58 ± 9.30 vs. 117.65 ± 9.27 mmHg; p < 0.05) and diastolic blood pressure (DBP) (81.77 ± 8.07 vs. 78.26 ± 8.92 mmHg; p < 0.05) compared with those without MASLD. Anthropometric measurements were also significantly greater among participants with MASLD, including body weight (70.7 ± 13.1 vs. 64.3 ± 14.8 kg; p < 0.05), waist circumference (89 ± 10 vs. 83 ± 10 cm; p < 0.05), hip circumference (99 ± 9 vs. 95 ± 8 cm; p < 0.05), body mass index (24.62 ± 3.30 vs. 22.60 ± 3.39 kg/m²; p < 0.001), and waist-to-hip ratio (0.90 ± 0.08 vs. 0.87 ± 0.08; p < 0.05).

Ultrasonographic assessment showed hepatic steatosis in 55.8% of the participants, including Grade I steatosis in 42.2%, Grade II in 12.3%, and Grade III in 1.3%, while 44.2% had no steatosis. FibroScan-based assessment demonstrated S0 in 81.8% of participants, whereas 3.9%, 9.1%, and 5.2% had S1, S2, and S3 steatosis, respectively. Liver stiffness assessment showed that 90.9% of participants were classified as F0-F1, while 6.5%, 1.9%, and 0.6% were classified as F2, F3, and F4, respectively (Figure 1).

**Figure 1:**
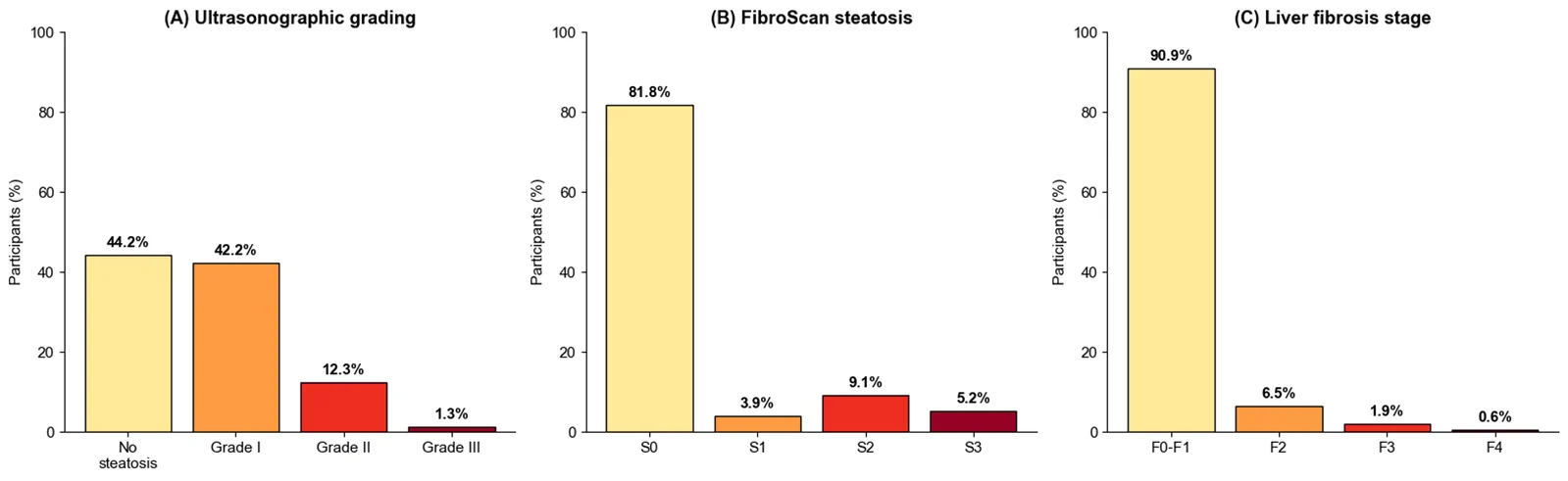
Prevalence and severity distribution of MASLD among the study participants Distribution of hepatic steatosis and liver fibrosis among study participants. (A) Ultrasonographic grading of hepatic steatosis. (B) FibroScan-based assessment of hepatic steatosis using controlled attenuation parameter (CAP). (C) Liver fibrosis staging based on liver stiffness measurement (LSM). Values are expressed as percentages of the total study population. Abbreviations: CAP, controlled attenuation parameter; LSM, liver stiffness measurement

The comparison of glycaemic and lipid parameters between participants with and without MASLD is presented in Table 2. Fasting serum insulin, fasting plasma glucose, glycated haemoglobin (HbA1c), HOMA-IR, and HOMA-β were comparable between the two groups, with no statistically significant differences observed (p > 0.05 for all). The median HOMA-IR was slightly higher among participants with MASLD than those without MASLD (2.33 (1.60-3.56) vs. 2.15 (1.43-2.93)), although the difference did not reach statistical significance (p = 0.228).

**Table 2:** Comparison of glycaemic and lipid parameters between participants with and without MASLD (N = 154) Data are presented as mean ± standard deviation, except for HOMA-IR, which is presented as median (interquartile range). Independent-samples Student’s t-test was used for normally distributed continuous variables, and the Mann–Whitney U test was used for HOMA-IR. P < 0.05 was considered statistically significant. *P < 0.05; **P < 0.001. Abbreviations: HOMA-β, homeostatic model assessment of β-cell function; HOMA-IR, homeostatic model assessment of insulin resistance; HDL, high-density lipoprotein; LDL, low-density lipoprotein

Parameter	No MASLD (n = 68)	MASLD (n = 86)	Reference range	Test statistic	P-value
Fasting serum insulin (µIU/mL)	9.91 ± 3.93	10.69 ± 3.88	2.6–24.9	t = −1.226	0.222
Fasting plasma glucose (mg/dL)	85.36 ± 8.64	85.75 ± 10.13	70–99	t = −0.251	0.802
HbA1c (%)	5.08 ± 0.31	5.11 ± 0.36	<5.7	t = −0.637	0.525
HOMA-IR	2.15 (1.43–2.93)	2.33 (1.60–3.56)	No universally established reference range	U = 2592.5	0.228
HOMA-β (%)	179.60 ± 116.96	191.18 ± 159.61	No universally established reference range	t = −0.501	0.617
Total cholesterol (mg/dL)	153.03 ± 27.50	166.95 ± 38.45	<200	t = −2.519	0.013*
HDL cholesterol (mg/dL)	42.55 ± 10.70	40.93 ± 9.64	≥40 (men); ≥50 (women)	t = 0.987	0.325
LDL cholesterol (mg/dL)	84.81 ± 27.39	104.25 ± 38.22	<100	t = −3.537	<0.001**
Triglycerides (mg/dL)	96.16 ± 49.66	115.30 ± 59.99	<150	t = −2.118	0.036*

By contrast, participants with MASLD demonstrated significantly higher levels of total cholesterol (166.95 ± 38.45 vs. 153.03 ± 27.50 mg/dL; p < 0.05), low-density lipoprotein cholesterol (104.25 ± 38.22 vs. 84.81 ± 27.39 mg/dL; p < 0.05), and triglycerides (115.30 ± 59.99 vs. 96.16 ± 49.66 mg/dL; p < 0.05) compared with those without MASLD. High-density lipoprotein cholesterol was marginally lower in the MASLD group; however, the difference was not statistically significant (40.93 ± 9.64 vs. 42.55 ± 10.70 mg/dL; p = 0.325).

Liver biochemical and transient elastography parameters

The comparison of liver biochemical and transient elastography parameters between participants with and without MASLD is presented in Table 3. Participants with MASLD had significantly higher serum aspartate aminotransferase (AST) (32.80 ± 16.05 vs. 26.36 ± 6.51 U/L; p < 0.05) and alanine aminotransferase (ALT) (41.07 ± 32.64 vs. 27.85 ± 13.78 U/L; p < 0.05) levels than those without MASLD. The median APRI score was slightly higher among participants with MASLD than those without MASLD (0.26 (0.20-0.40) vs. 0.24 (0.18-0.30)), and this difference was statistically significant (p < 0.05).

**Table 3:** Comparison of liver biochemical markers and transient elastography parameters between participants with and without MASLD (N = 154) Data are presented as mean ± standard deviation or median (interquartile range), as appropriate. Independent-samples Student’s t-test was used for AST, ALT, platelet count, and LSM; the Mann–Whitney U test was used for CAP and APRI because of their non-normal distributions. P < 0.05 was considered statistically significant. *P < 0.05; **P < 0.001. Abbreviations: CAP, controlled attenuation parameter; LSM, liver stiffness measurement; APRI, AST-to-platelet ratio index; AST, aspartate aminotransferase; ALT, alanine aminotransferase

Parameter	No MASLD (n = 68)	MASLD (n = 86)	Reference range / interpretation	Test statistic	P-value
AST (U/L)	26.36 ± 6.51	32.80 ± 16.05	0–40 U/L	t = −3.110	0.002*
ALT (U/L)	27.85 ± 13.78	41.07 ± 32.64	0–40 U/L	t = −3.125	0.002*
Platelet count (×10⁹/L)	218.57 ± 48.40	216.41 ± 54.07	150–450 ×10⁹/L	t = 0.259	0.796
CAP (dB/m)	180.50 (160.00–196.25)	218.00 (193.50–249.00)	S0: <248; S1: 248–267; S2: 268–279; S3: ≥280 dB/m	U = 1255.0	<0.001**
LSM (kPa)	3.98 ± 0.71	4.62 ± 1.26	F0–F4 fibrosis staging	t = −3.805	<0.001**
APRI	0.24 (0.18–0.30)	0.26 (0.20–0.40)	<0.5: low probability of significant fibrosis	U = 2384.5	<0.050*

Transient elastography demonstrated significantly greater hepatic steatosis in the MASLD group, with higher median controlled attenuation parameter (CAP) values (218.00 (193.50-249.00) vs. 180.50 (160.00-196.25); p < 0.001). Similarly, liver stiffness measurement (LSM) was significantly higher among participants with MASLD than those without MASLD (4.62 ± 1.26 vs. 3.98 ± 0.71 kPa; p < 0.001).

Point-biserial correlation analysis demonstrated significant positive correlations of MASLD status with SBP, DBP, weight, waist circumference, hip circumference, BMI, WHR, total cholesterol, LDL cholesterol, triglycerides, AST, ALT, APRI, CAP, and LSM. The strongest association was observed with CAP (r = 0.463, P < 0.001). MASLD status was not significantly correlated with age, pulse rate, height, fasting plasma glucose, fasting insulin, HbA1c, HOMA-IR, HOMA-β, HDL cholesterol, or platelet count (Table 4).

**Table 4:** Point-biserial correlations between MASLD status and clinical, anthropometric, metabolic, and hepatic parameters Note: Point-biserial correlation was used to assess associations between binary MASLD status and continuous variables. Two-tailed P < 0.05 was considered statistically significant. Abbreviations: APRI, AST-to-platelet ratio index; AST, aspartate aminotransferase; ALT, alanine aminotransferase; BMI, body mass index; CAP, controlled attenuation parameter; DBP, diastolic blood pressure; FPG, fasting plasma glucose; HDL, high-density lipoprotein; HOMA-β, homeostatic model assessment of β-cell function; HOMA-IR, homeostatic model assessment of insulin resistance; LDL, low-density lipoprotein; LSM, liver stiffness measurement; SBP, systolic blood pressure; WHR, waist-to-hip ratio

Parameter	Correlation coefficient (r)	95% CI	P-value
Age	0.033	−0.126 to 0.190	0.681
SBP	0.207	0.050 to 0.354	0.010
DBP	0.203	0.046 to 0.350	0.012
Pulse rate	0.055	−0.104 to 0.211	0.495
Height	0.054	−0.105 to 0.210	0.507
Weight	0.225	0.069 to 0.370	0.005
Waist circumference	0.290	0.138 to 0.429	<0.001
Hip circumference	0.201	0.044 to 0.348	0.012
BMI	0.291	0.139 to 0.429	<0.001
WHR	0.187	0.030 to 0.335	0.020
FPG	0.020	−0.139 to 0.178	0.802
Fasting insulin	0.099	−0.060 to 0.253	0.222
HbA1c	0.052	−0.107 to 0.208	0.525
HOMA-IR	0.010	−0.148 to 0.168	0.907
HOMA-β	0.041	−0.118 to 0.198	0.617
Total cholesterol	0.200	0.043 to 0.347	0.013
HDL cholesterol	−0.080	−0.235 to 0.079	0.325
LDL cholesterol	0.276	0.123 to 0.416	<0.001
Triglycerides	0.169	0.011 to 0.319	0.036
AST	0.245	0.090 to 0.388	0.002
ALT	0.246	0.091 to 0.389	0.002
Platelet count	−0.021	−0.179 to 0.138	0.796
APRI	0.211	0.055 to 0.357	0.009
CAP	0.463	0.329 to 0.579	<0.001
LSM	0.295	0.144 to 0.433	<0.001

The agreement between ultrasonographic and FibroScan-based classification of hepatic steatosis was evaluated using a 2 × 2 cross-tabulation (Table 5). Among the 154 participants, 66 were classified as having no steatosis by both ultrasonography and FibroScan, whereas 26 were classified as having steatosis by both methods. Thus, the two methods agreed in 92 participants, corresponding to an overall percentage agreement of 59.7%. However, 60 participants were classified as having steatosis by ultrasonography but not by FibroScan, while two participants were classified as having steatosis by FibroScan but not by ultrasonography. Cohen’s kappa was 0.251 (95% CI: 0.147-0.354), indicating fair agreement between the two methods. These findings demonstrate substantial discordance between ultrasonographic and CAP-based classification of hepatic steatosis in this cohort.

**Table 5:** Cross-tabulation of ultrasonographic and FibroScan-based classification of hepatic steatosis Values are presented as number of participants. Overall percentage agreement between ultrasonography and FibroScan-based steatosis classification was 59.7%. Cohen’s kappa (κ) was 0.251 (95% CI: 0.147–0.354), indicating fair agreement between the two methods. Abbreviations: MASLD, metabolic dysfunction-associated steatotic liver disease; S0, no steatosis by controlled attenuation parameter (CAP); S1–S3, hepatic steatosis by CAP

Ultrasonographic grading	FibroScan steatosis		Total, n
	Fibroscan:NO Steatosis (S0),n	Fibroscan: Steatosis (S1-3),n	
No MASLD	66	2	68
MASLD	60	26	86
Total	126	28	154

## Discussion

The present study evaluated the prevalence of MASLD and its association with clinical, anthropometric, metabolic, and hepatic parameters among apparently healthy young adults. More than half of the participants (55.8%) demonstrated ultrasonographic evidence of MASLD, highlighting a substantial burden of hepatic steatosis even in a relatively young and ostensibly healthy population. Participants with MASLD exhibited significantly higher body weight, BMI, waist circumference, hip circumference, waist-to-hip ratio, systolic and diastolic blood pressure, total cholesterol, low-density lipoprotein cholesterol, triglycerides, aminotransferase levels, CAP, LSM, and APRI scores than those without MASLD. Conversely, fasting plasma glucose, fasting insulin, HbA1c, HOMA-IR, and HOMA-β did not differ significantly between the two groups. These findings demonstrate that anthropometric adiposity measures and dyslipidaemia were significantly associated with MASLD in this cohort, whereas measures of glucose homeostasis did not differ significantly between groups. However, because of the cross-sectional design, the temporal relationship between these metabolic characteristics and hepatic steatosis cannot be established.

The observed prevalence of MASLD (55.8%) was higher than estimates reported for the general adult population globally and in India. Global meta-analyses suggest that MASLD affects approximately one-third of adults worldwide, while Indian studies have reported prevalence estimates ranging from 25% to 38%, depending on the study population and diagnostic modality used [3,7,8]. The higher prevalence observed in the present study is likely attributable to the hospital-based design and the inclusion of young medical students, a population potentially exposed to sedentary lifestyles, irregular dietary habits, academic stress, and reduced physical activity. Furthermore, ultrasonography is sensitive for detecting moderate-to-severe hepatic steatosis and may identify a greater proportion of asymptomatic individuals than routine clinical assessment. Collectively, these findings reinforce the growing burden of MASLD among young adults and emphasize the need for early screening in populations traditionally considered at low metabolic risk.

Participants with MASLD demonstrated significantly greater body weight, BMI, waist circumference, hip circumference, and waist-to-hip ratio than those without MASLD. These observations are consistent with previous studies demonstrating that both generalized and central adiposity are major determinants of hepatic fat accumulation [7-10,18]. Among South Asians, visceral adiposity frequently develops despite relatively modest BMI values, contributing to increased hepatic free fatty acid flux, adipose tissue dysfunction, and insulin resistance [9,11]. Waist circumference and waist-to-hip ratio are recognised as surrogate markers of visceral adiposity and have been shown to predict MASLD more accurately than BMI alone in Asian populations [22,23]. The significantly higher systolic and diastolic blood pressure observed among participants with MASLD further supports the close relationship between hepatic steatosis and the broader spectrum of cardiometabolic risk factors. Although blood pressure values remained largely within the normotensive range, these findings suggest that subtle cardiovascular alterations may already be present during the early stages of metabolic dysfunction.

An important finding of the present study was the presence of significant dyslipidaemia among participants with MASLD. Individuals with MASLD had significantly higher concentrations of total cholesterol, low-density lipoprotein cholesterol, and triglycerides, whereas high-density lipoprotein cholesterol was lower, although the difference did not reach statistical significance. These observations are consistent with the characteristic atherogenic lipid profile associated with hepatic steatosis and insulin resistance [12,18,25,26]. Hepatic triglyceride accumulation promotes increased very-low-density lipoprotein production and impaired lipid clearance, contributing to elevated circulating triglycerides and low-density lipoprotein cholesterol [12,25]. In contrast, fasting plasma glucose, fasting insulin, HbA1c, HOMA-IR, and HOMA-β were comparable between the two groups. Although insulin resistance is widely regarded as a central mechanism in MASLD pathogenesis [12-17], the absence of statistically significant differences in these indices may reflect the relatively young age of the participants and the early stage of metabolic dysfunction, before clinically detectable abnormalities in glucose metabolism become apparent. Similar findings have been reported in studies demonstrating that hepatic steatosis may precede overt hyperglycaemia and diabetes in otherwise healthy individuals [17,18].

Consistent with previous literature, participants with MASLD demonstrated significantly higher AST and ALT concentrations than those without MASLD, indicating early hepatocellular injury associated with hepatic fat accumulation [4,18]. Moreover, transient elastography revealed significantly higher CAP and LSM values among participants with MASLD, confirming greater hepatic steatosis and early fibrotic changes. Although the majority of participants exhibited no or minimal fibrosis, a small proportion had evidence of mild-to-moderate fibrosis, suggesting that structural liver changes may occur even in asymptomatic young adults. These findings are in agreement with current evidence supporting transient elastography as a reliable non-invasive method for quantifying hepatic steatosis and fibrosis and for identifying individuals at increased risk of progressive liver disease [5,21,27,28]. The significantly higher APRI scores observed in the MASLD group further support the presence of early hepatic injury, although APRI is primarily intended for fibrosis assessment and should be interpreted alongside imaging findings.

The findings of the present study have important clinical implications. The high prevalence of MASLD among apparently healthy young adults, together with its association with adverse anthropometric characteristics, dyslipidaemia, elevated liver enzymes, and early fibrotic changes, underscores the value of opportunistic screening in individuals with increased adiposity or metabolic risk factors, even in the absence of diabetes or clinically overt metabolic syndrome. Simple anthropometric measurements such as BMI and waist circumference, combined with routine biochemical testing and non-invasive imaging modalities including ultrasonography and transient elastography, may facilitate early identification of high-risk individuals. Early lifestyle modification focusing on weight reduction, dietary improvement, and regular physical activity has been shown to improve hepatic steatosis and reduce the long-term risk of type 2 diabetes, cardiovascular disease, and progressive liver fibrosis [4,18-20].

Recent Indian studies provide additional context for the present findings. Studies among young Indian adults and health-sciences students have demonstrated the presence of MASLD in relatively young populations, although differences in population characteristics and diagnostic methodology limit direct comparison with our cohort [30,31]. The multicentre MAP study, which used CAP and liver stiffness measurement across different Indian populations, further demonstrated substantial heterogeneity in MASLD prevalence and liver stiffness findings across populations [32]. These differences may reflect variations in age, metabolic risk profile, sampling strategy, and diagnostic modality. Emerging evidence regarding GLP-1 receptor agonists and their effects on obesity-related metabolic dysfunction and alcohol-related behaviours provides additional therapeutic context; however, this evidence is not directly comparable with the prevalence findings of the present study [33].

The present study has several strengths. It simultaneously evaluated anthropometric indices, metabolic parameters, insulin resistance, liver biochemistry, ultrasonographic findings, and transient elastography-derived measures of steatosis and liver stiffness in a cohort of apparently healthy young adults. The use of both conventional ultrasonography and transient elastography provided complementary non-invasive assessments of hepatic steatosis and liver stiffness. Nevertheless, several limitations should be considered. First, the cross-sectional design precludes establishing temporal or causal relationships between MASLD and the observed metabolic and hepatic characteristics. Second, the study was conducted at a single tertiary-care centre and included only undergraduate and postgraduate medical students; therefore, the findings may not be generalizable to the broader young adult population. Third, consecutive sampling in a hospital-based setting may have introduced selection bias. Fourth, ultrasonography and controlled attenuation parameter (CAP) assess hepatic steatosis using different techniques and thresholds, and the observed discordance between the two modalities should therefore be interpreted cautiously rather than assuming equivalence between their classifications. Fifth, liver biopsy was not performed because of the ethical and practical considerations associated with an invasive procedure in an apparently healthy population. Finally, multivariable analysis was not performed; therefore, the observed relationships represent unadjusted associations and should not be interpreted as independent predictors of MASLD.

## Conclusions

The present study demonstrates a high prevalence of ultrasonographically defined MASLD among apparently healthy young adults and identifies significant unadjusted associations with adiposity-related anthropometric measures, dyslipidaemia, liver enzymes, and non-invasive measures of hepatic steatosis and liver stiffness. However, the cross-sectional design, single-centre medical-student population, and absence of multivariable analysis limit causal inference, generalizability, and assessment of independent associations. Prospective multicentre studies with appropriate multivariable analyses are warranted to determine temporal relationships and independent risk factors for MASLD and its progression.
